# Metabolic Engineering of *Pseudomonas putida* KT2440 to Produce Anthranilate from Glucose

**DOI:** 10.3389/fmicb.2015.01310

**Published:** 2015-11-24

**Authors:** Jannis Kuepper, Jasmin Dickler, Michael Biggel, Swantje Behnken, Gernot Jäger, Nick Wierckx, Lars M. Blank

**Affiliations:** ^1^Institute of Applied Microbiology (iAMB), Aachen Biology and Biotechnology (ABBt), Rheinisch-Westfälische Technische Hochschule Aachen UniversityAachen, Germany; ^2^Bayer Technology Services GmbHLeverkusen, Germany; ^3^Bayer MaterialScience AGLeverkusen, Germany

**Keywords:** *Pseudomonas putida* KT2440, anthranilic acid, aromatic amino acid pathway, metabolic engineering, industrial biotechnology

## Abstract

The *Pseudomonas putida* KT2440 strain was engineered in order to produce anthranilate (oAB, *ortho*-aminobenzoate), a precursor of the aromatic amino acid tryptophan, from glucose as sole carbon source. To enable the production of the metabolic intermediate oAB, the *trpDC* operon encoding an anthranilate phosphoribosyltransferase (TrpD) and an indole-3-glycerol phosphate synthase (TrpC), were deleted. In addition, the chorismate mutase (*pheA*) responsible for the conversion of chorismate over prephenate to phenylpyruvate was deleted in the background of the deletion of *trpDC* to circumvent a potential drain of precursor. To further increase the oAB production, a feedback insensitive version of 3-deoxy-D-arabino-heptulosonate-7-phosphate synthase encoded by the *aro*G^*D146N*^ gene and an anthranilate synthase (*trpE^*S40F*^G*) were overexpressed separately and simultaneously in the deletion mutants. With optimized production conditions in a tryptophan-limited fed-batch process a maximum of 1.54 ± 0.3 g L^-1^ (11.23 mM) oAB was obtained with the best performing engineered *P. putida* KT2440 strain (*P. putida* Δ*trpDC* pSEVA234_*aro*G^*D146N*^_*trpE^*S40F*^G*).

## Introduction

Anthranilate (oAB, *ortho*-aminobenzoate) is an aromatic acid used as a platform chemical for the production of food ingredients (Raffensperger and Vogt, 1961), dyes, perfumes (Wiklund and Bergman, 2006), crop protection compounds (Askham, 1992; Yadav and Krishnan, 1998; Chambers et al., 2013), pharmaceutical compounds (Bahia et al., 2011; Shafiq et al., 2011; Haynes et al., 2012; Gao et al., 2013; Loque and Weniger, 2013; Walsh et al., 2013), and plastics such as nylon (Sun et al., 2013). It is currently produced in energy intensive chemical processes from petroleum-based precursors, like phthalamic acid (Klipper and Gripper, 1981; Berg, 2009). Furthermore, the production of the precursors and the production of oAB accumulate toxic byproducts, such as hypochlorite which is used with molar equivalency to oAB (Berg, 2009). Thus, there is a strong motivation to find alternative routes to produce platform chemicals, such as oAB, in green production processes from renewable resources in an environmental friendly way. In addition, the development and application of green production processes is accelerated by an environmental and political interest to be less dependent on fossil resources.

Biocatalysis using living microbes as catalysts is a well-established alternative for the production of chemicals. The aromatic biosynthesis pathway and the derived compounds of the aromatic acids, such as oAB, have been intensively studied in the last decades (Bongaerts et al., 2001; Ikeda, 2003; Kramer et al., 2003; Leuchtenberger et al., 2005; Pittard and Yang, 2008). Microbial production of oAB with engineered *Escherichia coli* strains was reported by Balderas-Hernandez et al. (2009) followed by further publications on oAB-derived compounds such as catechol and muconic acid (Sun et al., 2013; Averesch and Krömer, 2014; Balderas-Hernandez et al., 2014; Jaeger et al., 2015). To enable oAB production in *E. coli*, Balderas-Hernandez et al. (2009, 2014) inserted a point mutation in the oAB phosphoribosyl transferase domain (*trpD*), whereas Sun et al. (2013) used the Keio collection deletion strain *E. coli* BW25113 Δ*trp::kan* to prevent the conversion of oAB to tryptophan. Additional targets to increase the production of oAB in *E. coli*, for example the overexpression of feedback insensitive variants of the 3-deoxy-D-arabino-heptulosonate-7-phosphate (DAHP) synthase and the anthranilate synthase unit (*trpE^*S40F*^G*) were investigated. A maximum titer of 14 g L^-1^ oAB was reported growing the engineered strains in complex medium containing 30 g L^-1^ yeast extract (Balderas-Hernandez et al., 2009).

Here, we present the first attempt of microbial production of oAB from glucose as sole carbon source with an engineered *P seudomonas putida* KT2440 strain. Due to its versatile metabolism and low nutritional requirements *P. putida* is an efficient production strain for various industrial relevant products (Tiso et al., 2014). In addition its high biomass yield, high growth rate, and low maintenance demand fulfill the rigorous demands of industrial biotechnology (Poblete-Castro et al., 2012). A broad portfolio of *P. putida* biocatalysts for bulk chemicals such as phenol (Wierckx et al., 2005), *p*-hydroxystyrene (Verhoef et al., 2009), *p***-**hydroxybenzoate (Verhoef et al., 2007), rhamnolipids (Wittgens et al., 2011), polyhydroxyalkanoates (PHA; Wang et al., 2011), and (S)-styrene oxide (Blank et al., 2008) demonstrate the great potential of this species as a flexible cell factory for the production of chemicals in industrial biotechnology. In addition, *P. putida* strains have the capability to withstand various chemical stresses such as a second phase of toluene, octanol, or styrene (Heipieper and de Bont, 1994; Dominguez-Cuevas et al., 2006; Blank et al., 2008), as well as oxidative stress (Chavarria et al., 2013) and reduced water activity (Hallsworth et al., 2003), and thus providing a promising and versatile chassis for the production of toxic compounds such as oAB.

To ensure industrially relevant oAB production conditions a full, markerless deletion of *trpDC* was performed in *P. putida*, facilitated by the fact that in contrast to *E. coli* the *trpEG* and *trpDC* genes are encoded by separate open reading frames. Additionally the production of oAB was realized on glucose as sole carbon source, avoiding the addition of high amounts of complex media components such as yeast extract. A maximum titer of 1.54 ± 0.3 g L^-1^ (11.23 mM) oAB was obtained with the best performing engineered *P. putida* KT2440 strain (*P. putida* Δ*trpDC* pSEVA234_*aroG^*D146N*^_trpE^*S40F*^G*) in tryptophan-limited fed-batch fermentations with glucose as sole carbon source.

## Materials and Methods

### Strains and Plasmids

The deletion of *trpDC* and *pheA* were performed by a clean and markerless deletion method described by Martinez-Garcia and de Lorenzo (2011) resulting in two knock out strains *P. putida KT2440 trpDC* and *P. putida* KT2440 *trpDC pheA*. To obtain the knockout vectors pEMG_Δ*trpDC* and pEMG_Δ*pheA* were obtained via a standard restriction and ligation approach and were transformed into chemical competent *E. coli* DH5α (according to Choi et al., 2006) via electroporation. The 800-bp flanks upstream (TS1) and downstream (TS2) of the gene of interest (*trpDC* and *pheA*) were amplified by PCR using a Pfu polymerase (New England Biolabs) with the primers listed in **Table 1**.

**Table 1:** Primer sequences.

Name	DNA sequence	*T*_m_ (°C)
**TS1 ΔtrpDC**		
JK034f	agggataacagggtaatctgaatTCGTCAGCAAACTCTTGATG	61.6
JK035r	tttgactcgagGTTCGATCCTTAACGGCG	61.6
**TS2 ΔtrpDC**		
JK036f	aggatcgaacctcgagTCAAATGAAGCCGGCGTT	66.1
JK037r	cctgcaggtcgactctagaggatccTCGAACCAAGGTGCTACCG	66.1
**TS1 ΔpheA**		
JK038f	attcgagctcggtacccggggatccACTACATCGAAACCGGCATC	61.8
JK039r	ctgaactcgagTCAGCCATGCTCCTTCTC	61.8
**TS2 ΔpheA**		
JK040f	gcatggctgactcgagTTCAGGGGCCTTGGGGCT	70.2
JK041r	tagaagcttgcatgcctgcaggCAGTGAGTCGACCAGGCCAAAG	70.2

TS1 and TS2 were fused in a SOEing-PCR using Pfu polymerase according to Horton (1995). The backbone (pEMG) and the fused SOEing-PCR fragment were digested with *BamHI* and *EcoRI* for the deletion of *trpDC* and with *BamHI* and *SbfI* for the deletion of *pheA*. The digested backbones, TS1, and TS2 were purified (High Pure PCR Product Purification Kit, Roche), ligated with a T4 DNA ligase (Thermo Fisher Scientific) and transformed into chemical competent *E. coli* DH5α (according to Choi et al., 2006) via electroporation. Constructs were verified by restriction analysis and sequencing, resulting in pEMG_Δ*trpDC* and pEMG_Δ*pheA*. Genome integration of the knockout constructs into the *P. putida* strains was performed via tri**-**parental mating according to Ditta et al. (1980) using *E. coli* HB101 pRK2013 as the helper strain and facilitated as described in Zobel et al. (2015) where the three mating strains were streaked one above the other on a LB plate. The resulting strains were transformed with the plasmid expressing the I*Sce*-I endonuclease (pSW-I; according to Choi et al., 2006). Induction with 3-methylbenzoate was omitted due to the leaky expression of the I*Sce*-I nuclease. Successful construction of the knockout strains was verified via restriction, PCR and Sanger sequencing.

The feedback insensitive overexpression constructs were obtained via a standard restriction and ligation approach as described above using *BamHI* and *EcoRI* for *aroG^*D146N*^* and *BamHI* for *trpE^*S40F*^G*. pSEVA234 (Silva-Rocha et al., 2013), which contains an IPTG inducible lacI^Q^-P_trc_ expression system, was used as backbone. The genes *aroG^D146N^* (Kikuchi et al., 1997; Albermann et al., 2014) and *trpE^*S40F*^G* (Kwak et al., 1999) were synthesized at Eurofins Genomics. A summary of the used and constructed plasmids and of the engineered strains is shown in **Table 2**. All primers were purchased at Eurofins Genomics and all restriction enzymes at Thermo Fisher Scientific.

**Table 2:** Summary of plasmids and strains used in this study.

	Description	Reference
**Plasmids**		
pEMG	Km^R^, *ori*R6K, *lacZ*a with two flanking I-*Sce*I sites	Martinez-Garcia and de Lorenzo, 2011
pSEVA234	Km^R^, *oriBBR1*, *lacI^q^*-*Ptrc*	Silva-Rocha et al., 2013
pSW-I	Ap^R^, *ori*RK2, *xylS*, *Pm*→*I-Sce*I	Martinez-Garcia and de Lorenzo, 2011
pRK2013	Km^R^, *ori*RK2, *ori*ColE1	Figurski et al., 1979
pEMG_Δ*trpDC*	*trpDC* deletion plasmid	This work
pEMG_Δ*pheA*	*pheA* deletion plasmid	This work
pSEVA234_*trpE^*S40F*^G*	*trpE^*S40F*^G* expression plasmid	This work
pSEVA234_*aroG^**D146N**^*	*aroG^*D146N*^* expression plasmid	This work
pSEVA234_*aroG^*D146N*^*_*trpE^*S40F*^G*	*aroG^*D146N*^*-*trpE^*S40F*^G* expression plasmid	This work
**Strain**		
*Psuedomonas putida* KT2440	Wild-type strain derived of *P. putida* mt-2 cured of the pWW0 plasmid	Bagdasarian et al., 1981
*Escherichia coli* DH5α	*supE44*, D*lacU169 (*f*80 lacZ*D*M15), hsdR17 (rk-mk*+*), recA1, endA1, thi1, gyrA, relA*	Hanahan, 1985
*E. coli* DH5α aaapir	aaapir phage lysogen of DH5α	De Lorenzo Lab collection
*E. coli* HB101 pRK2013	Sm^R^, *hsdR-M*+, *pro, leu, thi, recA*, Km^R^, *ori*RK2, *ori*ColE1	Figurski et al., 1979
*E. coli* DH5α aaapir pEMG	Plasmid carrier strain	Martinez-Garcia and de Lorenzo, 2011
*E. coli* DH5α aaapir pSW-I	Plasmid carrier strain	Martinez-Garcia and de Lorenzo, 2011
*E. coli* DH5α aaapir pEMG_Δ*trpDC*	Plasmid carrier strain	This work
*E. coli* DH5α aaapir pEMG_Δ*pheA*	Plasmid carrier strain	This work
*E. coli* DH5α pSEVA234_*trpE^*S40F*^G*	Plasmid carrier strain	This work
*E. coli* DH5α pSEVA234_*aroG^*D146N*^*	Plasmid carrier strain	This work
*E. coli* DH5α pSEVA234_*aroG^*D146N*^*_*trpE^*S40F*^G*	Plasmid carrier strain	This work
*P. putida* KT2440 pSEVA234_*trpE^*S40F*^G*	oAB production strain	This work
*P. putida* KT2440 pSEVA234_*aroG^*D146N*^*	oAB production strain	This work
*P. putida* KT2440 pSEVA234_*aroG^*D146N*^*_*trpE^*S40F*^G*	oAB production strain	This work
*P. putida* KT2440 Δ*trpDC*	oAB production strain	This work
*P. putida* KT2440 Δ*trpDC* pSEVA234_*trpE^*S40F*^G*	oAB production strain	This work
*P. putida* KT2440 Δ*trpDC* pSEVA234_*aroG^*D146N*^*	oAB production strain	This work
*P. putida* KT2440 Δ*trpDC* pSEVA234_*aroG^*D146N*^*_*trpE^*S40F*^G*	oAB production strain	This work
*P. putida* KT2440 Δ*trpDC* Δ*pheA*	oAB production strain	This work
*P. putida* KT2440 Δ*trpDC* Δ*pheA* pSEVA234_*trpE^*S40F*^G*	oAB production strain	This work
*P. putida* KT2440 Δ*trpDC* Δ*pheA* pSEVA234_*aroG^*D146N*^*	oAB production strain	This work
*P. putida* KT2440 Δ*trpDC* Δ*pheA* pSEVA234_*aroG^*D146N*^*_*trpE^*S40F*^G*	oAB production strain	This work

### Cultivation Conditions

For cloning and maintenance processes, *E. coli* strains and *P. putida* strains were cultivated at 37 and 30°C, respectively, in LB medium supplemented with or without kanamycin (50 mg L^-1^) or ampicillin (100 mg L^-1^ for *E. coli* and 500 mg L^-1^ for *P. putida*), and/or with 1.5% (w/v) agar as needed.

Auxotrophies (tryptophan and phenylalanine) of the gene deletion mutants were verified on solid mineral medium plates (Wierckx et al., 2005) with 1.5% (w/v) agar, 20 mM glucose with and without 1 mM tryptophan, and/or 1 mM phenylalanine supplementation. Alternatively 1 mM phenylpyruvate was used instead of phenylalanine.

Batch-wise oAB production was performed in 500 mL shake flasks at 30°C and 200 rpm in 50 mL mineral medium as described in Wierckx et al. (2005) with 20 mM glucose (unless stated differently), 50 mg L^-1^ kanamycin, and 1 mM IPTG, supplemented with either 0.1 or 0.05 mM tryptophan and 1 mM phenlypyruvate for the Δ*pheA* strains. Two additional 20 mM glucose pulses were added after 10 and 24 h unless stated differently.

Tryptophan-limited fed-batch conditions were realized in controlled bioreactors (BioFlo 110 or BioFlo 115, Eppendorf / New Brunswick Scientific) with a starting volume of 400 mL. The initial fermentation medium consisted of mineral medium with 50 mM glucose, a twofold phosphate buffer concentration, a threefold (NH_4_)_2_SO_4_ concentration, a onefold trace element solution, 1 mM IPTG, 50 mM kanamycin, and 0.1 mM tryptophan. After the initial batch phase, the feed was switched on at a rate of 2 mL h^-1^ consisting of a mixed solution of 1 M glucose and 0.5 mM (glucose to tryptophan molar ratio of 2,000:1) or 1 mM (glucose to tryptophan molar ratio of 1,000:1) tryptophan. To compensate for the increasing biomass concentrations the 1 mM tryptophan feed was increased to 6 mL h^-1^. The fermentations were performed at 30°C, with 500–1,200 rpm agitation (dO_2_ regulated agitation cascade with a lower limit of 35%), with 1 vvm headspace aeration of compressed air. The pH was regulated to pH = 7 with 2 M KOH and 4 M H_2_SO_4_.

### Analytics

The biomass concentration was measured with a spectrophotometer (Ultrospec 10, GE Healthcare Life Sciences). In this device the OD_600_ correlates to cell dry weight (CDW): 1 OD_600_ = 0.505 g_CDW_ L^-1^. The samples taken during cultivation were centrifuged at 13,300 rpm for 3 min and stored at -20°C for further analysis. To follow the consumption of the glucose and derivatives (gluconate and 2**-**ketogluconate) by the *P. putida* KT2440 strains, a Beckman HPLC equipped with an organic acid resin column (polystyrol**-**divinylbenzol copolymer, PS-DVB: 300 × 8.0 mm, CS-Chromatographie) was used with 5 mM H_2_SO_4_ as eluent at a flow of 0.8 mL h^-1^ for 11 min at 75°C. Detection was realized with an UV detector at a wavelength of 210 nm and a RI detector. The oAB production was analyzed with a reverse phase column (LiChrosorb 100 RP-18, 250 × 4 mm, Merck), at a flow of 1.2 mL h^-1^ [pump gradient of H_2_O + 0.1% TFA (pump A) and of MeOH (pump B): 0–2 min 90% A, 2–12 min gradient 0–90% A, 12–14 min 0% A, 14–15 min gradient 0–90% A, and 15–16 min 90% A] at 30°C. Detection was realized with an UV detector at a wavelength of 257 nm and a RI detector.

## Results and Discussion

### Metabolic Engineering of oAB Production Strains

**FIGURE 1:**
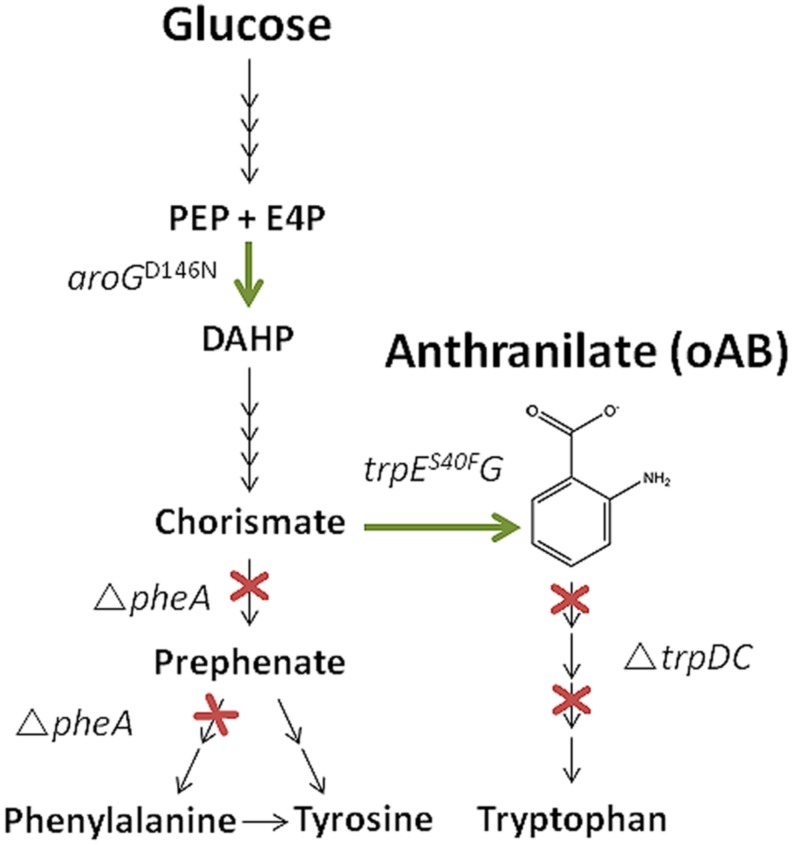
**Schematic oAB production pathway including the metabolic engineering targets investigated in this study (overexpression of *aroG^*D146N*^trpE^*S40F*^G* and deletion of *trpDC* and *pheA)*.** PEP, phosphoenolpyruvate; E4P, erythrose-4-phosphate; DAHP, 3-Deoxy-D-arabinoheptulosonate-7-phosphate.

In order to establish oAB production in *P. putida*, the *trpDC* and *pheA* genes were knocked out using the I-*Sce*I-based pEMG system (Martinez-Garcia and de Lorenzo, 2011). Disruption of the *trpDC* genes, which encode an anthranilate phosphoribosyltransferase (TrpD) and an indole-3-glycerol phosphate synthase (TrpC), leads to a tryptophan auxotrophy and enables the accumulation of oAB (**Figure 1**). Disruption of the *pheA* gene, which encodes a bifunctional chorismate mutase/prephenate dehydratase enzyme responsible for the first two steps of the synthesis of phenylalanine and tyrosine, possibly increases the metabolic flux toward oAB by reducing the drain on its primary precursor chorismate (Zhao et al., 2011). Contrary to other established production hosts, the *pheA* deletion only requires phenylalanine to complement growth since *P. putida* can convert phenylalanine to tyrosine (Molina-Henares et al., 2009). The corresponding auxotrophies were verified on mineral medium plates (**Table 3**).

**Table 3:** Auxotrophy supplementation of *P. putida* KT2440 Δ*trpDC* and Δ*pheA* knockouts.

Name	Supplementation^a^	Growth
*P. putida* KT2440	None	+
	trp	+
	phe	+
	trp + phe	+
*P. putida* KT2440 Δ*trpDC*	None	-
	Trp	+
*P. putida* KT2440 Δ*trpDC* Δ*pheA*	None	-
	Trp	-
	trp + phe	+
	trp + pp	+

*^a^Mineral medium plates with 20 mM glucose supplemented with 1 mM tryptophan (trp), phenylalanine (phe), or phenylpyruvate (pp)*.

In the knockout process, the deletion of *pheA* could only be obtained by supplementation with phenylpyruvate. The final step of the knockout procedure (induction of the double strand break) should theoretically yield a one-to-one ratio of wildtype to knockout allele. However, selection on LB- or LB medium with phenylalanine resulted in the wildtype allele only, even after testing >1,000 colonies either by PCR or by screening for phenylalanine auxotrophy. This may be attributed to the ability of *P. putida* to degrade phenylalanine and tyrosine. Possibly, supplementation with phenylpyruvate instead of phenylalanine reduced the induction of genes encoding the phenylalanine and tyrosine catabolic pathway (Arias-Barrau et al., 2004), facilitating the successful isolation of the knockout strain. The final *pheA* knockout auxotroph could be complemented with phenylalanine in mineral medium. However, in this case a severe negative effect on the fitness of the mutant caused by the deletion of *pheA* was observed. Therefore, all subsequent Δ*pheA* complementation were done with phenylpyruvate.

To further optimize the production of oAB in *P. putida*, feedback insensitive pSEVA234-based (Silva-Rocha et al., 2013) overexpression constructs for *trpE^*S40F*^G* and *aroG^*D146N*^*, or both genes in one operon structure, were transformed to the respective mutants under the IPTG-inducible LacI^Q^-P_trc_ system. These genes encode feedback insensitive variants of anthranilate synthase and 3-deoxy-D-arabino-heptulosonate-7-phosphate (DHAP) synthase, respectively, and are known to enhance oAB production in *E. coli* (Balderas-Hernandez et al., 2009, 2014; Sun et al., 2013). **Figure 1** shows the exemplarily oAB production pathway and gives on overview over the metabolic engineering targets investigated in this study.

### Evaluation of oAB Production Strains in Shake Flasks

**FIGURE 2:**
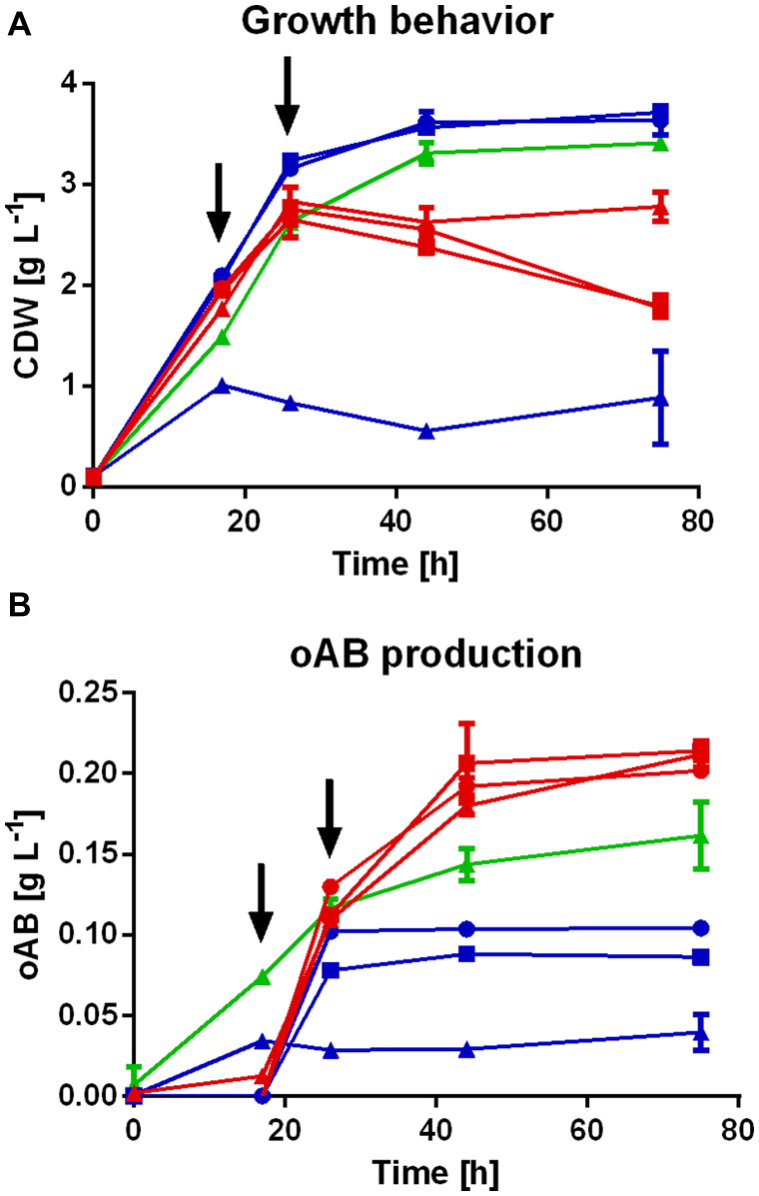
**oAB production profiles of various *P. putida* KT2440 strains in shake flasks.** Biomass growth **(A)** and oAB production **(B)** of the following *Pseudomonas putida* KT2440 strains in an initial screening experiment: 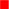, Δ*trpDC* pSEVA234_*trpE^*S40F*^G*; 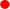, Δ*trpDC* pSEVA234_*aroG^*D146N*^*; 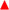, Δ*trpDC* pSEVA234_*aroG^*D146N*^_trpE^*S40F*^G*; 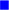, Δ*trpDC* Δ*pheA* pSEVA234_*trpE^*S40F*^G*; 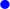, Δ*trpDC* Δ*pheA* pSEVA234_*aroG^*D146N*^*; 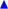, Δ*trpDC* Δ*pheA* pSEVA234_*aroG^*D146N*^_trpE^*S40F*^G*; 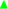, pSEVA234_*aroG^*D146N*^_trpE^*S40F*^G*. All cultures were performed in mineral medium with 20 mM initial glucose concentration and addition of tryptophan and/or phenylpyruvate as described above. The arrows indicate the addition of glucose to a concentration of 20 mM.

**FIGURE 3:**
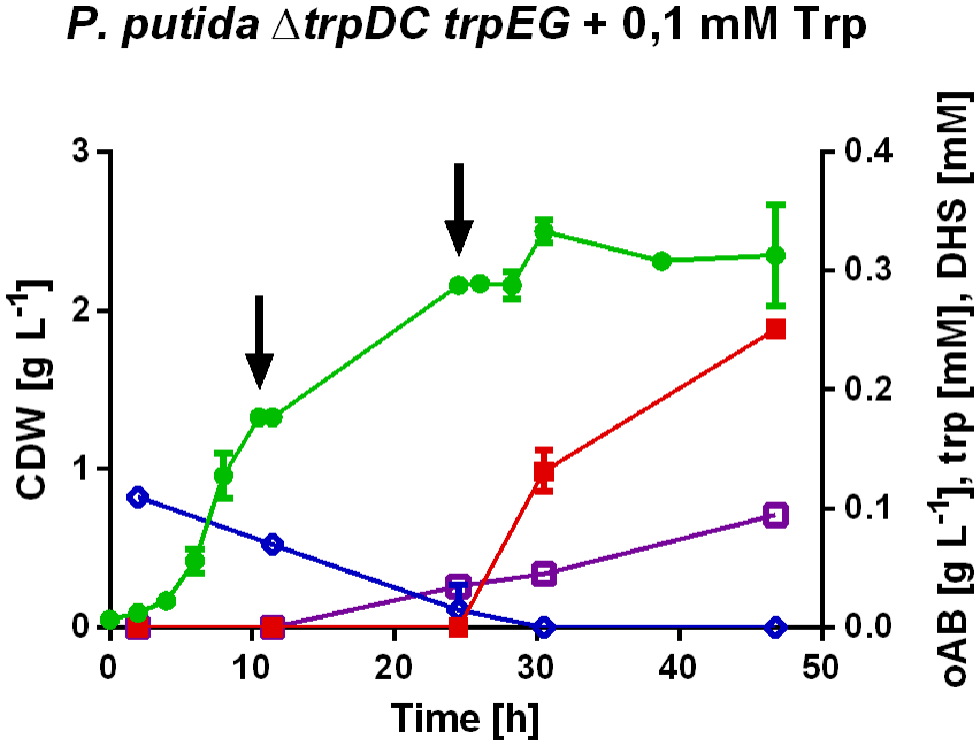
**Detailed profiles of 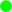, cell dry weight; 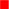, oAB; 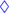, tryptophan (trp); 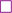, dehydroshikimate (DHS) concentrations of the best selected strain *P.putida* KT2440 Δ*trpDC* pSEVA234_*trpE^*S40F*^G* generated under slightly optimized production conditions.** All cultures were performed in mineral medium with 20 mM initial glucose concentration and addition of tryptophan and/or phenylpyruvate as described above. The arrows indicate the addition of glucose to a concentration of 20 mM.

The *P. putida* strains engineered for the production of oAB (listed in **Table 2**) were initially assessed in shake flasks (**Figures 2A,B**) and under slightly optimized production conditions a maximum titer of 0.25 ± 0.004 g L^-1^ (1.83 mM) oAB with glucose as sole carbon source was achieved (**Figure 3**). The three Δ*trpDC* strains bearing either *trpE^*S40F*^G*, *aroG^*D146N*^* or both, have shown no significant differences in maximal oAB titers, although the onset of production was earlier in the *P. putida* KT2440 Δ*trpDC* pSEVA234_*aroG^*D146N*^*_*trpE^*S40F*^G*. Interestingly, oAB production was also observed with *P. putida* KT2440 pSEVA234_*aroG^*D146N*^*_*trpE^*S40F*^G* (without *trpDC* deletion) while no tryptophan was secreted, although the maximal titer was lower than that of the Δ*trpDC* strains. This can be explained by the transcriptional repression of *trp* genes by tryptophan through the TrpR repressor (Maurer and Crawford, 1971; Wierckx et al., 2008). Likely, an increase of intracellular tryptophan caused repression of the native *trp* genes, leading to anthranilate accumulation due to the heterologous expression of *trpE^*S40F*^G*.

A strong connection was observed between tryptophan limitation and oAB production. When supplementing the auxotrophic strains with tryptophan the oAB production was induced only upon depletion of the added tryptophan. Strains expressing both *aroG^*D146N*^* and *trpE^*S40F*^G* produced oAB at earlier time points compared to strains with only one of the feedback insensitive genes, indicating somewhat alleviated feedback insensitivity to tryptophan (**Figure 2**). With the non-auxotrophic *P. putida* pSEVA234_*aroG^*D146N*^*_*trpE^*S40F*^G*, where a supplementation with tryptophan was not required, oAB titers were significantly higher at earlier time points, indicating no inhibition by tryptophan. However, the final oAB titers were 34% lower compared to the *P. putida* Δ*trpDC* strains, indicating a positive effect of the deletion of *trpDC*.

As indicated above, a clear negative effect of the deletion of *pheA* on the growth behavior was observed. Whereas *P. putida* KT2440 pSEVA234_*trpE^*S40F*^G* and *P. putida* KT2440 Δ*trpDC* pSEVA234_*trpE^*S40F*^G* were able to grow up to 3.6 g L^-1^ CDW and 2.4 g L^-1^ CDW, respectively; *P.putida* KT2440 Δ*trpDC* Δ*pheA* pSEVA234_*trpE^*S40F*^G* only reached a maximal CDW concentration of 1.4 g L^-1^ after 10 hours when supplemented with 1 mM phenylpyruvate. Further addition of glucose and/or tryptophan could neither initiate growth to higher CDW concentrations, nor did it improve oAB production. Normal growth was only fully rescued when supplementing high amounts of phenylpyruvate (≥5 mM) which would make the overall process highly uneconomical. Additionally, final oAB titers were still 49% lower than with the Δ*trpDC* strains, indicating a negative effect of the *pheA* deletion for the production of oAB in this organism. Thus, the most promising strain engineered for the production of oAB is *P. putida* Δ*trpDC* pSEVA234_*aroG^*D146N*^*_*trpE^*S40F*^G* as it reached high titers of oAB and showed reduced sensitivity to tryptophan.

Dehydroshikimate, a metabolic intermediate of the shikimate pathway and thus a precursor of oAB, accumulated as a by-product in all strains engineered for oAB production in shake flasks, indicating shikimate dehydrogenase as a likely bottleneck (**Figure 3**). This hypothesis is also supported by the transcriptome data sets of Wierckx et al. (2009) and Verhoef et al. (2010) showing upregulated 3-dehydroquinate and dehydroshikimate genes in the analyzed phenol and *p*-hydroxybenzoate production strains obtained by a fluoro-analog mutant screening.

### Production of oAB in Controlled Bioreactors

**FIGURE 4:**
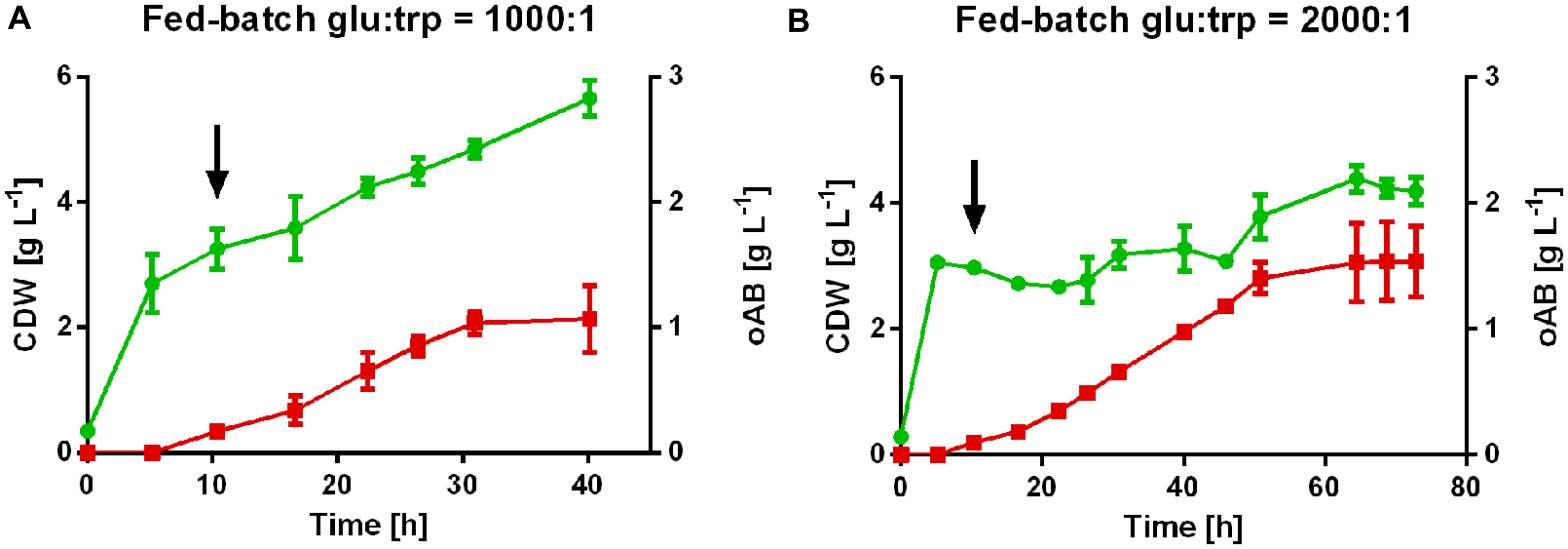
**Tryptophan-limited fed-batch cultures of *P. putida* KT2440 Δ*trpDC* pSEVA234_*aroG^*D146N*^_trpE^*S40F*^G* in controlled bioreactors with different feeding approaches. (A)** Feed molar ratio of glucose to tryptophan 1,000:1. **(B)** Feed molar ratio of glucose-to-tryptophan 2,000:1. 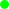, cell dry weight; 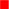, oAB concentration. The arrows indicate the start of the feed after the initial batch phase.

The potential of *P. putida* Δ*trpDC* pSEVA234_*aroG^*D146N*^*_*trpE^*S40F*^G* to produce oAB was further assessed in tryptophan-limited fed batch cultures to circumvent the observed inhibition by tryptophan and maximize final oAB titers. A glucose-to-tryptophan molar ratio of 400:1 was estimated for biomass growth alone based on the initial shake flask experiments. Therefore, two different feeding approaches with a molar ratio of glucose to tryptophan of 1,000:1 and 2,000:1 were used to ensure a tryptophan limitation without excessive accumulation of glucose or its derivatives. Under these conditions a maximal titer of 1.54 ± 0.3 g L^-1^ oAB was reached from glucose as sole carbon source using the 2,000:1 feed (**Figure 4**). The higher ratio of glucose to tryptophan led to a more severe growth limitation, with CDW increasing only marginally during the production of oAB. In contrast, the 1,000:1 feed enabled more biomass growth at the cost of oAB production, leading to a final product titer of 1.0 ± 0.07 g L^-1^. The product per substrate yield (based on consumed carbon source) for both conditions is relatively similar at 3.6 ± 0.5% (g/g) for the 1,000:1 feed and 3.5 ± 0.5% (g/g) for the 2,000:1 feed. oAB levels increased fairly linearly until the production stopped abruptly. Since the level of oAB produced is well below growth-inhibiting concentrations for *P. putida* (data not shown), oAB production is most likely stopped due to product inhibition, a known phenomenon for the production of aromatics (Gibson and Pittard, 1968; Wierckx et al., 2008; Rodriguez et al., 2014). This product inhibition likely takes place at the level of the anthranilate synthase. Indeed, the anthranilate synthase complex of other organisms is already inhibited by oAB concentrations in the micromolar range (Cordaro et al., 1968; Henderson et al., 1970; Francis et al., 1978). The oAB titers obtained with *P. putida* KT2440 in this study are about 10-fold lower than those achieved by Balderas-Hernandez et al. (2009). The difference can most likely be attributed to the supplementation of 30 g/L yeast extract by these researchers, which can provide oAB precursors and increases the general stress tolerance of microorganisms. This apparent positive effect of yeast extract on oAB production should be further investigated in order to elucidate the responsible components.

In the initial batch phase, the 50 mM glucose were entirely consumed for the production of biomass. Tryptophan limiting conditions were confirmed by HPLC analysis throughout the fermentation (<0.1 mM). Carbon source, either as glucose or as gluconate and 2-ketogluconate, were constantly present during the feed phase at total concentrations between 0.4 and 8.6 g L^-1^. In some fermenters, a prolonged incubation led to a decrease of oAB concentrations over time. Possibly, polymerization of oAB and/or its conversion products occurred.

## Conclusion

Microbial production of oAB under industrial relevant conditions from glucose as sole carbon source was achieved in *P. putida* KT2440 via the biosynthesis pathway of aromatic amino acids. A strong connection between a tryptophan limitation and oAB production was observed even with strains bearing feedback insensitive overexpression constructs of *aroG^*D146N*^* and *trpE^*S40F*^G*. Under tryptophan limiting fed-batch conditions, a maximum titer of 1.54 ± 0.3 g L^-1^ oAB was achieved with *P. putida* KT2440 Δ*trpDC* pSEVA234_*aroG^*D146N*^*_*trpE^*S40F*^G*. This final achieved concentration is in the same range as other aromatics produced by *P. putida* strains (Nijkamp et al., 2005, 2007; Verhoef et al., 2007); although the titer is lower than that of previously published works with *E. coli* (Balderas-Hernandez et al., 2009). However, the supplementation with yeast extract was avoided and oAB was only produced from glucose. In addition, to ensure long term strain stability, one of the main requirements in industrial biotechnology, a stable and markerless deletion of the genes responsible for the conversion of oAB towards tryptophan (*trpDC*) was used. Nevertheless, the oAB titer and yield reached with *P. putida* KT2440 are below those which are required to realize an industrial feasible process. Further improvement is required, e.g., by more in-depth metabolic engineering (e.g., overexpression of *tkt*: Balderas-Hernandez et al., 2009) as well as by *in situ* product removal to alleviate product inhibition. Further research on the mechanism of product inhibition of oAB production could also lead to additional metabolic engineering targets to improve microbial oAB production.

## Conflict of Interest Statement

The authors declare that the research was conducted in the absence of any commercial or financial relationships that could be construed as a potential conflict of interest.

## References

[B1] Albermann C. Weiner M. Trondle J. Weuster-Botz D. Sprenger G. A. (2014). Utilization of organophosphate:phosphate antiporter for isotope-labeling experiments in *E. coli*. *FEMS Microbiol. Lett.* 361 52–61. 10.1111/1574-6968.12612 25273627

[B2] Arias-Barrau E. Olivera E. R. Luengo J. M. Fernandez C. Galan B. Garcia J. L. (2004). The homogentisate pathway: a central catabolic pathway involved in the degradation of L-phenylalanine, L-tyrosine, and 3-hydroxyphenylacetate in *Pseudomonas putida*. *J. Bacteriol.* 186 5062–5077. 10.1128/JB.186.15.5062-5077.2004 15262943 PMC451635

[B3] Askham L. R. (1992). “Efficacy of methyl anthranilate as a bird repellent on cherries, blueberries and grapes,” in *Proceeding of the 15th Vertebrate Pest Conference*, eds Borrecco J. E. Marsh R. E. (Davis, CA: University of California), 137–141.

[B4] Averesch N. J. H. Krömer J. O. (2014). Tailoring strain construction strategies for muconic acid production in *S. cerevisiae* and *E. coli*. *Metab. Eng. Commun.* 1 19–28. 10.1016/j.meteno.2014.09.001 PMC8193250 34150501

[B5] Bagdasarian M. Lurz R. Ruckert B. Franklin F. C. Bagdasarian M. M. Frey J. (1981). Specific-purpose plasmid cloning vectors. II. Broad host range, high copy number, RSF1010-derived vectors, and a host-vector system for gene cloning in *Pseudomonas*. *Gene* 16 237–247. 10.1016/0378-1119(81)90080-9 6282695

[B6] Bahia M. S. Gunda S. K. Gade S. R. Mahmood S. Muttineni R. Silakari O. (2011). Anthranilate derivatives as TACE inhibitors: docking based CoMFA and CoMSIA analyses. *J. Mol. Model.* 17 9–19. 10.1007/s00894-010-0695-7 20349256

[B7] Balderas-Hernandez V. E. Sabido-Ramos A. Silva P. Cabrera-Valladares N. Hernandez-Chavez G. Baez-Viveros J. L. (2009). Metabolic engineering for improving anthranilate synthesis from glucose in *Escherichia coli*. *Microb. Cell Fact.* 8:19 10.1186/1475-2859-8-19 PMC2671490 19341482

[B8] Balderas-Hernandez V. E. Trevino-Quintanilla L. G. Hernandez-Chavez G. Martinez A. Bolivar F. Gosset G. (2014). Catechol biosynthesis from glucose in *Escherichia coli* anthranilate-overproducer strains by heterologous expression of anthranilate 1,2-dioxygenase from *Pseudomonas aeruginosa* PAO1. *Microb. Cell Fact.* 13:136 10.1186/s12934-014-0136-x PMC4190458 25281236

[B9] Berg C. (2009). *Treatment of Aqueous Liquids and the Preparation of Anthranilic Acid.* US 20090171116 A1.

[B10] Blank L. M. Ionidis G. Ebert B. E. Buhler B. Schmid A. (2008). Metabolic response of *Pseudomonas putida* during redox biocatalysis in the presence of a second octanol phase. *FEBS J.* 275 5173–5190. 10.1111/j.1742-4658.2008.06648.x 18803670

[B11] Bongaerts J. Kramer M. Muller U. Raeven L. Wubbolts M. (2001). Metabolic engineering for microbial production of aromatic amino acids and derived compounds. *Metab. Eng.* 3 289–300. 10.1006/mben.2001.0196 11676565

[B12] Chambers A. H. Evans S. A. Folta K. M. (2013). Methyl anthranilate and gamma-decalactone inhibit strawberry pathogen growth and achene Germination. *J. Agric. Food Chem.* 61 12625–12633. 10.1021/jf404255a 24328200

[B13] Chavarria M. Nikel P. I. Perez-Pantoja D. De Lorenzo V. (2013). The Entner-Doudoroff pathway empowers *Pseudomonas putida* KT2440 with a high tolerance to oxidative stress. *Environ. Microbiol.* 15 1772–1785. 10.1111/1462-2920.12069 23301697

[B14] Choi K. H. Kumar A. Schweizer H. P. (2006). A 10-min method for preparation of highly electrocompetent *Pseudomonas aeruginosa* cells: application for DNA fragment transfer between chromosomes and plasmid transformation. *J. Microbiol. Methods* 64 391–397. 10.1016/j.mimet.2005.06.001 15987659

[B15] Cordaro J. C. Levy H. R. Balbinder E. (1968). Product inhibition of anthranilate synthetase in *Salmonella* typhimurium. *Biochem. Biophys. Res. Commun.* 33 183–189. 10.1016/0006-291X(68)90765-1 4881047

[B16] Ditta G. Stanfield S. Corbin D. Helinski D. R. (1980). Broad host range DNA cloning system for gram-negative bacteria: construction of a gene bank of *Rhizobium meliloti*. *Proc. Natl. Acad. Sci. U.S.A.* 77 7347–7351. 10.1073/pnas.77.12.7347 7012838 PMC350500

[B17] Dominguez-Cuevas P. Gonzalez-Pastor J. E. Marques S. Ramos J. L. De Lorenzo V. (2006). Transcriptional tradeoff between metabolic and stress-response programs in *Pseudomonas putida* KT2440 cells exposed to toluene. *J. Biol. Chem.* 281 11981–11991. 10.1074/jbc.M509848200 16495222

[B18] Figurski D. H. Meyer R. J. Helinski D. R. (1979). Suppression of Co1E1 replication properties by the Inc P-1 plasmid RK2 in hybrid plasmids constructed in vitro. *J. Mol. Biol.* 133 295–318. 10.1016/0022-2836(79)90395-4 395312

[B19] Francis M. M. Vining L. C. Westlake D. W. (1978). Characterization and regulation of anthranilate synthetase from a chloramphenicol-producing streptomycete. *J. Bacteriol.* 134 10–16. 306386 10.1128/jb.134.1.10-16.1978 PMC222211

[B20] Gao X. Jiang W. Jimenez-Oses G. Choi M. S. Houk K. N. Tang Y. (2013). An iterative, bimodular nonribosomal peptide synthetase that converts anthranilate and tryptophan into tetracyclic asperlicins. *Chem. Biol.* 20 870–878. 10.1016/j.chembiol.2013.04.019 23890005 PMC3728708

[B21] Gibson F. Pittard J. (1968). Pathways of biosynthesis of aromatic amino acids and vitamins and their control in microorganisms. *Bacteriol. Rev.* 32 465–492. 4884716 PMC413161

[B22] Hallsworth J. E. Heim S. Timmis K. N. (2003). Chaotropic solutes cause water stress in *Pseudomonas putida*. *Environ. Microbiol.* 5 1270–1280. 10.1111/j.1462-2920.2003.00519.x 14641573

[B23] Hanahan D. (1985). “Techniques for transformation of *E. coli*,” in *DNA Cloning: A Practical Approach*, ed. Glover D. M. (Oxford: IRL Press), 109–135.

[B24] Haynes S. W. Gao X. Tang Y. Walsh C. T. (2012). Assembly of asperlicin peptidyl alkaloids from anthranilate and tryptophan: a two-enzyme pathway generates heptacyclic scaffold complexity in asperlicin E. *J. Am. Chem. Soc.* 134 17444–17447. 10.1021/ja308371z 23030663 PMC3500603

[B25] Heipieper H. J. de Bont J. A. M. (1994). Adaptation of *Pseudomonas putida* S12 to ethanol and toluene at the level of fatty acid composition of membranes. *Appl. Environ. Microbiol.* 60 4440–4444. 7811084 10.1128/aem.60.12.4440-4444.1994 PMC202003

[B26] Henderson E. J. Nagano H. Zalkin H. Hwang L. H. (1970). The anthranilate synthetase-anthranilate 5-phosphoribosylpyrophosphate phosphoribosyltransferase aggregate. Purification of the aggregate and regulatory properties of anthranilate synthetase. *J. Biol. Chem.* 245 1416–1423. 4315598

[B27] Horton R. M. (1995). PCR-mediated recombination and mutagenesis. SOEing together tailor-made genes. *Mol. Biotechnol.* 3 93–99. 10.1007/BF02789105 7620981

[B28] Ikeda M. (2003). Amino acid production processes. *Adv. Biochem. Eng. Biotechnol.* 79 1–35. 12523387 10.1007/3-540-45989-8_1

[B29] Jaeger G. Magnus J. Moussa A. S. Olf G. Lolli G. Behnken S. (2015). *Recombinant Strain Producing O-Aminobenzoate and Fermentative Production of Aniline from Renewable Resources Via 2-Aminobenzoic Acid*. Google Patents 2015124687.

[B30] Kikuchi Y. Tsujimoto K. Kurahashi O. (1997). Mutational analysis of the feedback sites of phenylalanine-sensitive 3-deoxy-D-arabino-heptulosonate-7-phosphate synthase of *Escherichia coli*. *Appl. Environ. Microbiol.* 63 761–762. 9023954 10.1128/aem.63.2.761-762.1997 PMC168366

[B31] Klipper G. Gripper J. (1981). *Continuous Preparation of Anthranilic Acid.* US 4276433 A.

[B32] Kramer M. Bongaerts J. Bovenberg R. Kremer S. Muller U. Orf S. (2003). Metabolic engineering for microbial production of shikimic acid. *Metab. Eng.* 5 277–283. 10.1016/j.ymben.2003.09.001 14642355

[B33] Kwak J. H. Hong K. W. Lee S. H. Hong J. H. Lee S. Y. (1999). Identification of amino acid residues involved in feedback inhibition of the anthranilate synthase in *Escherichia coli*. *J. Biochem. Mol. Biol.* 32 20–24.

[B34] Leuchtenberger W. Huthmacher K. Drauz K. (2005). Biotechnological production of amino acids and derivatives: current status and prospects. *Appl. Microbiol. Biotechnol.* 69 1–8. 10.1007/s00253-005-0155-y 16195792

[B35] Loque D. Weniger A. G. E. (2013). *Host Cells and Methods for Producing Cinnamoyl Anthranilate and Analogs Thereof.* US 20130078683 A1.

[B36] Martinez-Garcia E. de Lorenzo V. (2011). Engineering multiple genomic deletions in Gram-negative bacteria: analysis of the multi-resistant antibiotic profile of *Pseudomonas putida* KT2440. *Environ. Microbiol.* 13 2702–2716. 10.1111/j.1462-2920.2011.02538.x 21883790

[B37] Maurer R. Crawford I. P. (1971). New regulatory mutation affecting some of the tryptophan genes in *Pseudomonas putida*. *J. Bacteriol.* 106 331–338. 5573729 10.1128/jb.106.2.331-338.1971 PMC285101

[B38] Molina-Henares M. A. Garcia-Salamanca A. Molina-Henares A. J. De La Torre J. Herrera M. C. Ramos J. L. (2009). Functional analysis of aromatic biosynthetic pathways in *Pseudomonas putida* KT2440. *Microb. Biotechnol.* 2 91–100. 10.1111/j.1751-7915.2008.00062.x 21261884 PMC3815424

[B39] Nijkamp K. Van Luijk N. De Bont J. A. Wery J. (2005). The solvent-tolerant *Pseudomonas putida* S12 as host for the production of cinnamic acid from glucose. *Appl. Microbiol. Biotechnol.* 69 170–177. 15824922 10.1007/s00253-005-1973-7

[B40] Nijkamp K. Westerhof R. G. Ballerstedt H. De Bont J. A. Wery J. (2007). Optimization of the solvent-tolerant *Pseudomonas putida* S12 as host for the production of p-coumarate from glucose. *Appl. Microbiol. Biotechnol.* 74 617–624. 10.1007/s00253-006-0703-0 17111138

[B41] Pittard J. Yang J. (2008). Biosynthesis of the aromatic amino acids. *EcoSal Plus* 1 1–39. 10.1128/ecosalplus.3.6.1.8 26443741

[B42] Poblete-Castro I. Becker J. Dohnt K. Dos Santos V. M. Wittmann C. (2012). Industrial biotechnology of *Pseudomonas putida* and related species. *Appl. Microbiol. Biotechnol.* 93 2279–2290. 10.1007/s00253-012-3928-0 22350258

[B43] Raffensperger S. P. Vogt R. D. (1961). *Stabilization of Grape Flavored Soft Drink Mixes Containing Methyl Anthranilate.* US 3005715 A.

[B44] Rodriguez A. Martinez J. A. Flores N. Escalante A. Gosset G. Bolivar F. (2014). Engineering *Escherichia coli* to overproduce aromatic amino acids and derived compounds. *Microb. Cell. Fact.* 13:126 10.1186/s12934-014-0126-z PMC4174253 25200799

[B45] Shafiq M. Zia-Ur-Rehman M. Khan I. U. Arshad M. N. Khan S. A. (2011). Synthesis of novel anti-bacterial 2,1-benzothiazine 2,2-dioxides derived from methyl anthranilate. *J. Chil. Chem. Soc.* 56 527–531. 10.4067/S0717-97072011000100001

[B46] Silva-Rocha R. Martinez-Garcia E. Calles B. Chavarria M. Arce-Rodriguez A. De Las Heras A. (2013). The standard european vector architecture (SEVA): a coherent platform for the analysis and deployment of complex prokaryotic phenotypes. *Nucleic Acids Res.* 41 D666–D675. 10.1093/nar/gks1119 23180763 PMC3531073

[B47] Sun X. Lin Y. Huang Q. Yuan Q. Yan Y. (2013). A novel muconic acid biosynthesis approach by shunting tryptophan biosynthesis via anthranilate. *Appl. Environ. Microbiol.* 79 4024–4030. 10.1128/AEM.00859-13 23603682 PMC3697559

[B48] Tiso T. Wierckx N. Blank L. M. (2014). *Non-pathogenic Pseudomona*s as *Platform for Industrial Biocatalysis* Singapore: Pan Stanford Publishing.

[B49] Verhoef S. Ballerstedt H. Volkers R. J. M. De Winde J. H. Ruijssenaars H. J. (2010). Comparative transcriptomics and proteomics of p-hydroxybenzoate producing *Pseudomonas putida* S12: novel responses and implications for strain improvement. *Appl. Environ. Microbiol.* 87 679–690. 10.1007/s00253-010-2626-z PMC2874742 20449741

[B50] Verhoef S. Ruijssenaars H. J. De Bont J. A. Wery J. (2007). Bioproduction of p-hydroxybenzoate from renewable feedstock by solvent-tolerant *Pseudomonas putida* S12. *J. Biotechnol.* 132 49–56. 10.1016/j.jbiotec.2007.08.031 17900735

[B51] Verhoef S. Wierckx N. Westerhof R. G. De Winde J. H. Ruijssenaars H. J. (2009). Bioproduction of p-hydroxystyrene from glucose by the solvent-tolerant bacterium *Pseudomonas putida* S12 in a two-phase water-decanol fermentation. *Appl. Environ. Microbiol.* 75 931–936. 10.1128/AEM.02186-08 19060171 PMC2643573

[B52] Walsh C. T. Haynes S. W. Ames B. D. Gao X. Tang Y. (2013). Short pathways to complexity generation: fungal peptidyl alkaloid multicyclic scaffolds from anthranilate building blocks. *ACS Chem. Biol.* 8 1366–1382. 10.1021/cb4001684 23659680 PMC3796173

[B53] Wang H. H. Zhou X. R. Liu Q. Chen G. Q. (2011). Biosynthesis of polyhydroxyalkanoate homopolymers by *Pseudomonas putida*. *Appl. Microbiol. Biotechnol.* 89 1497–1507. 10.1007/s00253-010-2964-x 21046374

[B54] Wierckx N. J. Ballerstedt H. De Bont J. A. Wery J. (2005). Engineering of solvent-tolerant *Pseudomonas putida* S12 for bioproduction of phenol from glucose. *Appl. Environ. Microbiol.* 71 8221–8227. 10.1128/AEM.71.12.8221-8227.2005 16332806 PMC1317433

[B55] Wierckx N. J. P. Ballerstedt H. De Bont J. A. M. De Winde J. H. Ruijssenaars H. J. Wery J. (2008). Transcriptome analysis of a phenol-producing *Pseudomonas putida* S12 construct: genetic and physiological basis for improved production. *J. Bacteriol.* 190 2822–2830. 10.1128/JB.01379-07 17993537 PMC2293262

[B56] Wierckx N. Ruijssenaars H. J. De Winde J. H. Schmid A. Blank L. M. (2009). Metabolic flux analysis of a phenol producing mutant of *Pseudomonas putida* S12: verification and complementation of hypotheses derived from transcriptomics. *J. Biotechnol.* 143 124–129. 10.1016/j.jbiotec.2009.06.023 19560494

[B57] Wiklund P. Bergman J. (2006). The chemistry of anthranilic acid. *Curr. Organ. Synth.* 3 379–402. 10.2174/157017906777934926

[B58] Wittgens A. Tiso T. Arndt T. T. Wenk P. Hemmerich J. Muller C. (2011). Growth independent rhamnolipid production from glucose using the non-pathogenic *Pseudomonas putida* KT2440. *Microb. Cell Fact.* 10:80 10.1186/1475-2859-10-80 PMC3258213 21999513

[B59] Yadav G. D. Krishnan M. S. (1998). An ecofriendly catalytic route for the preparation of perfumery grade methyl anthranilate from anthranilic acid and methanol. *Organ. Process. Res. Dev.* 2 86–95. 10.1021/op980074j

[B60] Zhao Z. J. Zou C. Zhu Y. X. Dai J. Chen S. Wu D. (2011). Development of L-tryptophan production strains by defined genetic modification in *Escherichia coli*. *J. Ind. Microbiol. Biotechnol.* 38 1921–1929. 10.1007/s10295-011-0978-8 21541714

[B61] Zobel S. Benedetti I. Eisenbach L. De Lorenzo V. Wierckx N. Blank L. M. (2015). A Tn7-based device for calibrated heterologous gene expression in *Pseudomonas putida*. *ACS Synth. Biol.* 10.1021/acssynbio.5b00058 [Epub ahead of print]. 26133359

